# Best Practices for Germicidal Ultraviolet-C Dose Measurement for N95 Respirator Decontamination

**DOI:** 10.6028/jres.126.020

**Published:** 2021-10-20

**Authors:** Alisha Geldert, Halleh B. Balch, Anjali Gopal, Alison Su, Samantha M. Grist, Amy E. Herr

**Affiliations:** 1 Department of Bioengineering, University of California, Berkeley, Berkeley, CA 94720, USA; 2 The University of California, Berkeley – University of California, San Francisco Graduate Program in Bioengineering, Berkeley, CA 94720, USA; 3 Department of Physics, University of California, Berkeley, Berkeley, CA 94720, USA; 4 N95DECON Consortium, University of California, Berkeley, Berkeley, CA 94720, USA; * These authors contributed equally; # Corresponding author

**Keywords:** COVID-19, decontamination, dose measurement, germicidal ultraviolet irradiation, N95 respirator, personal protective equipment, ultraviolet-C

## Abstract

Ultraviolet-C (UV-C) decontamination holds promise in combating the coronavirus disease 2019 pandemic, particularly with its potential to mitigate the N95 respirator shortage. Safe, effective, and reproducible decontamination depends critically on UV-C dose, yet dose is frequently measured and reported incorrectly, which results in misleading and potentially harmful protocols. Understanding best practices in UV-C dose measurement for N95 respirator decontamination is essential to the safety of medical professionals, researchers, and the public. Here, we outline the fundamental optical principles governing UV-C irradiation and detection, as well as the key metrics of UV-C wavelength and dose. In particular, we discuss the technical and regulatory distinctions between UV-C N95 respirator decontamination and other applications of germicidal UV-C, and we highlight the unique considerations required for UV-C N95 respirator decontamination. Together, this discussion will inform best practices for UV-C dose measurement for N95 respirator decontamination during crisis-capacity conditions.

## 1 Introduction

The coronavirus disease 2019 (COVID-19) pandemic led to severe shortages of N95 filtering facepiece respirators, which are essential personal protective equipment (PPE) for healthcare professionals worldwide.^1^1 “N95” is a filter class designation of the U.S. National Institute of Occupational Safety and Health (NIOSH). It is applied to respirators that are at least 95% efficient at filtering NaCl aerosols with particle sizes of mean diameter 75 nm ± 20 nm (NIOSH Procedure No. TEB-APR-STP-0059, 13 December 2019). In response, the U.S. Centers for Disease Control and Prevention (CDC) issued guidelines for decontamination and reuse of N95 respirators as a crisis-capacity strategy and identified ultraviolet-C (UV-C) germicidal irradiation as one of the most promising methods for primary decontamination [1]. UV-C plays an important role in infection control across the medical industry, but, due to the complex geometry and material properties of N95 respirators, the UV-C measurement considerations for N95 respirator decontamination differ substantially from more established applications of germicidal UV-C. Safe and effective UV-C decontamination depends critically on (1) the spectral overlap between the emission spectrum of the light source and the wavelengths capable of inactivating the pathogen (i.e., the action spectrum), and (2) the amount of energy that is delivered to the pathogen (fluence, often described as dose^2^2 Not all the energy incident on a substrate is absorbed. While dose is almost always used in the germicidal UV-C literature to describe the energy incident on the material being decontaminated, dose can also refer to the total amount of absorbed (not incident) energy in other contexts. The most accurate technical term to describe the total incident UV-C energy (in units of J/cm^2^) on a surface is fluence [2]. However, to align with the germicidal UV-C literature, here we choose to use the term “dose” to describe total incident energy on an N95 respirator. Similarly, while fluence rate is a more technically accurate term to describe the radiant power (in units of W/cm^2^) irradiating a sample from all directions [2], here we use the term irradiance to align with the decontamination literature.). However, accurately measuring and reporting these characteristics for UV-C N95 respirator decontamination systems can be complicated, and measurement standards targeting the unique challenges of complex, multimaterial N95 respirators remain in development.

Accurate measurements of UV-C dose are central both for verifying that decontamination systems are operating within specification and for reproducible reporting. UV-C dose measurements provide a key link in the translation of effective and reproducible decontamination protocols across different communities, from UV-C device manufacturers and researchers to infection-control staff implementing UV-C N95 respirator decontamination. In this paper, we highlight key measurement considerations for researchers, engineers, and clinical staff who are evaluating and implementing UV-C–based decontamination of N95 respirators. First, we highlight the technical and regulatory context for UV-C N95 respirator decontamination; second, we discuss the science behind UV-C decontamination, highlighting the central importance of both wavelength and dose in viral inactivation; third, we examine techniques and common pitfalls in UV-C dose measurement; and finally, we outline best practices that help to avoid these pitfalls.

## 2 UV-C for N95 Respirator Decontamination

**Fig. 1:**
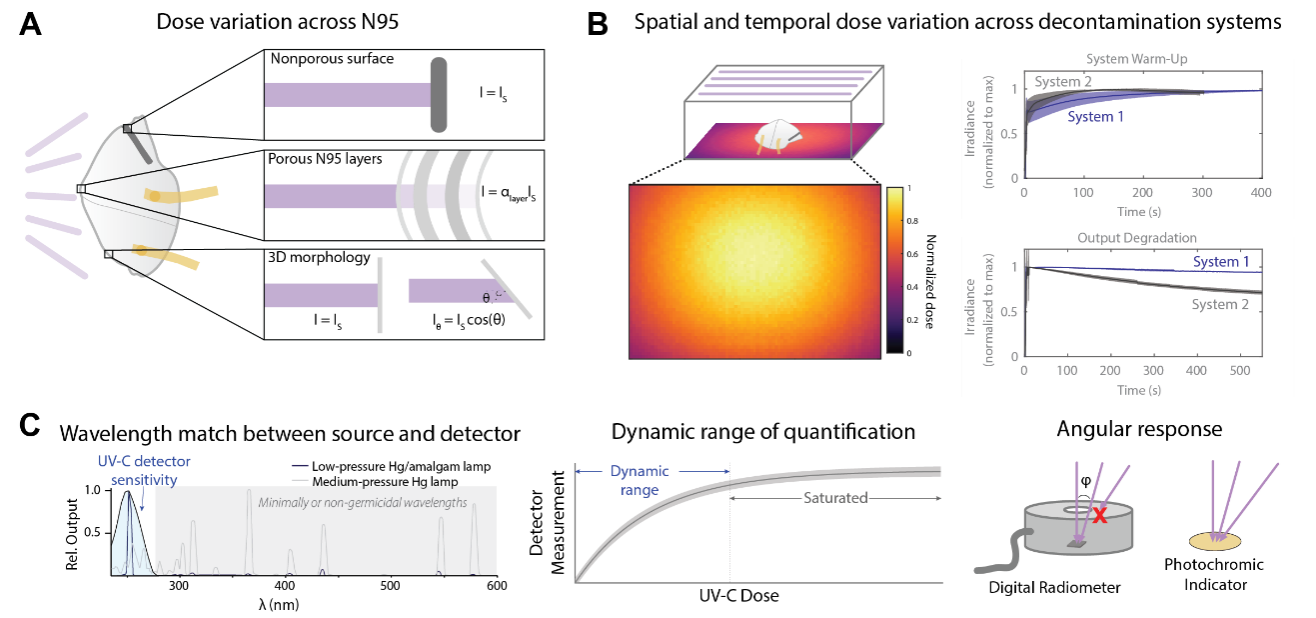
Factors affecting UV-C dose distribution and measurement for N95 respirator decontamination. (A) Factors affecting UV-C dose applied to the N95 respirator. Sloped surfaces and attenuation by the N95 layers reduce received UV-C dose. Received UV-C intensity (I) is reduced from the intensity normally incident on the top surface (I_s_) by a layer-dependent attenuator factor (α_layer_) and by a factor of the cosine of the angle of incidence (θ). (B) Factors affecting UV-C dose distribution within a decontamination system. UV-C irradiance can vary spatially and temporally. (C) Key specifications of UV-C detectors, including wavelength specificity, dynamic range, and angular response (ϕ denotes the radiometer field-of-view angle). Figure is adapted with permission from Su *et al*. [14].

UV-C radiation is widely used as a secondary technique for decontamination of air [3], water [4], and nonporous surfaces [5]. Until April 2021, CDC guidance [1] and hospital protocols [6] indicated that UV-C was used during the COVID-19 pandemic as a primary and stand-alone decontamination method for N95 respirators under crisis-capacity conditions [7]. As a primary decontamination technique, the application of UV-C to N95 respirators requires specific consideration of the complex geometry, porous multimaterial electret layers, and filtration central to N95 respirator function. For example, UV-C radiation is heavily attenuated when passing through non–UV-C-transparent and scattering materials; dose received at interior layers may be orders of magnitude lower than the applied dose at the outer surface of the N95 respirator (Fig. 1A) [8]. UV-C attenuation through the porous layers requires special consideration to ensure that the dose received at all contaminated layers within the respirator is sufficient for decontamination [8]. Consequently, decontamination of porous materials can require 100× higher applied dose at the surface than that required for nonporous surfaces with low surface roughness [9, 10], but excessive doses can reduce respirator function [11]. The electrostatic respirator filter material is also damaged by chemical disinfectants such as ethanol [12], limiting the use of some primary healthcare surface disinfectants. Furthermore, the complex three-dimensional geometry of N95 respirators can result in the received dose varying several-fold across a single N95 respirator [13, 14] and about twenty-fold across different N95 respirators within one decontamination system [14], with received dose strongly dependent on the incident angle of UV-C irradiation

(Fig. 1A–B) [15].

Due to the technical challenges and additional considerations required for implementing UV-C decontamination for N95 respirators, federal guidelines for UV-C decontamination of N95 respirators remain in development [16]. For example, the CDC has assessed the impact of several UV-C N95 respirator decontamination systems on the fit and filtration of specific N95 respirator models, but the assessment “is not to determine the effectiveness of the decontamination procedure at killing the pathogenic microorganism” [17]. The U.S. Food and Drug Administration (FDA) guidelines emphasize that while the FDA regulates UV-C sources, the lack of clear and standardized manufacturer data on wavelength, duration, and associated dose of UV-C radiation required to inactivate severe acute respiratory syndrome coronavirus 2 (SARS-CoV-2), the strain of coronavirus that causes COVID-19, presents an outstanding challenge [18]. In addition, the FDA allows previously approved devices to be extended to SARS-CoV-2 inactivation [19]. However, the FDA requires previously approved devices to submit Emergency Use Authorizations (EUAs) and 510(k) when adapted to new applications including decontamination of N95 respirators and other single-use PPE [15]. A 510(k) is a premarket submission made to the FDA to demonstrate that a device to be marketed is as safe and effective as a legally marketed device. For example, a steam sterilization device with prior 510(k) clearance for sterilization of other materials in healthcare settings required an FDA-issued EUA before it was approved for N95 respirator decontamination [20]. While the FDA has issued numerous EUAs for devices implementing the other two PPE decontamination methods (moist heat and vaporous hydrogen peroxide) [20, 21] identified promising by the CDC for crisis-capacity conditions, as of January 2021, only one limited EUA has been issued for the use of UV-C to reduce bioburden on one N95 respirator model [22].

Despite this context, the accessibility and relatively low cost of UV-C sources have led to widespread implementation of UV-C irradiation for N95 respirator decontamination in both research [12, 23, 24] and medical [6] environments. Decontamination system specifications depend on technical measurement factors, such as the wavelengths emitted, the wavelengths detected, the type and position of UV-C detector, and the method of analysis. Reports of UV-C–based decontamination of N95 respirators often fail to report the parameters necessary to ensure validation and reproducibility despite using diverse types of UV-C sources and different measurement devices. To accurately describe, evaluate, and reproduce UV-C decontamination protocols, parameters such as type, number, and location of UV-C sources, orientation and position of both N95(s) and UV-C detector(s) relative to UV-C source(s), models of N95 respirator and UV-C detector, decontamination chamber specifications (e.g., reflectivity), and other details of dose quantification (Appendix), are needed. Omitting these parameters for the source, target, or detector when reporting decontamination procedures substantially limits validation and reproducibility. In addition, standards for measurement are currently limited, which impedes comparison of UV-C sources and detectors [25]. In particular, minimum reporting standards for systems claiming UV-C decontamination of N95 respirators are urgently needed to facilitate comparison and critical evaluation. Here, we provide an overview of UV-C measurement fundamentals to inform the development of measurement and reporting standards for UV-C N95 respirator decontamination systems.

## 3 Key Germicidal UV-C Specifications: Wavelength and Dose

Not all wavelengths of UV radiation offer sufficient germicidal efficacy for N95 respirator decontamination. Absorbed germicidal UV-C radiation (200 nm to 280 nm) inactivates pathogens by promoting photochemical reactions that damage proteins and genomic material [26, 27]. Distinct wavelengths have different microbe-specific germicidal efficacy, a relationship represented in what is known as the action spectrum of a microbe. The overlap between the action spectrum and the UV-C source emission spectrum will determine the efficiency of germicidal action, with higher efficiency when the overlap is greater. For many pathogens, there is a peak in the action spectrum at the absorption maximum of genomic material, around 260 nm. While research into the germicidal action spectrum of SARS-CoV-2 is ongoing, a working assumption is that the action spectrum will be similar to that of viral analogues with similar structure that exhibit a peak near 260 nm [27, 28]. Germicidal UV-C radiation sources emit close to this maximum, such as the narrow emission around 254 nm from low-pressure mercury (Hg) lamps commonly used as germicidal sources. The relative efficacy of emerging monochromatic and polychromatic UV-C sources is also an area of active research, highlighting the importance of rigorous measurement and reporting to facilitate accurate comparison of sources with different emission spectra. While shorter wavelengths within the UV-C range (~200 nm to 220 nm) can have higher germicidal efficacy [27], these wavelengths may be more strongly attenuated by the multiple N95 layers, requiring confirmation of dose and viral inactivation on interior layers. Longer-wavelength UV radiation (>280 nm), such as UV-B and UV-A in sunlight, has substantially lower germicidal activity [29] and has not been shown to decontaminate porous materials such as N95 respirators. While UV-B (280 nm to 320 nm) can photochemically damage nucleic acids, UV-B is orders of magnitude less efficient than UV-C wavelengths [29] due to reduced overlap with the absorption spectrum of nucleic acids. While UV-A (320 nm to 400 nm) can generate reactive oxygen species to contribute to pathogen inactivation (particularly in water) [30, 31], UV-A is generally not considered to be germicidal [27]. Because absorption by the multiple porous N95 layers causes N95 respirator decontamination to require about 100× higher applied dose [8] as compared to more common applications (e.g., air, water, nonporous surface decontamination), UV-A and UV-B likely have insufficient germicidal efficacy to be feasible for N95 respirator decontamination.

Efficacy of germicidal UV-C also depends critically on dose. Studies on other coronaviruses and influenza viruses indicate that 254 nm UV-C doses (from a low-pressure mercury UV-C source) of at least 1.0 J/cm^2^ at the N95 respirator surface can lead to ≥99.9% viral inactivation on most N95 respirator models [9, 32]. Preliminary studies using both UV-C light-emitting diodes (LEDs) and mercury lamps have found that UV-C doses of at least 1.5 J/cm^2^ are required to yield ≥99.9% inactivation of SARS-CoV-2 on some N95 respirator models [12, 24], and research on SARS-CoV-2 inactivation on N95 respirators is ongoing. On the other hand, studies indicate that doses over 120 J/cm^2^ can cause respirator degradation [11]. Because it is infeasible to measure UV-C dose delivered to viral particles embedded in the interior layers of the respirator during a decontamination cycle, the dose required for pathogen inactivation or degradation is typically reported in terms of dose applied at the respirator surface. However, because UV-C transmission through N95 respirator layers is dependent on the N95 model [8], the minimum dose applied at the N95 surface for pathogen inactivation throughout all N95 layers will differ from model to model. These examples underscore the importance of accurate measurement and reporting of UV-C wavelength and dose when using germicidal UV-C for effective and reproducible decontamination of N95 respirators.

## 4 Critical UV-C Source and Detector Metrics

Applying sufficient UV-C dose to N95 respirators can make—or break—effective decontamination [33]. While measurement of pathogen inactivation is the most direct way of verifying decontamination efficacy on N95 respirators, this approach is time- and resource-intensive. It is largely infeasible to perform pathogen inactivation assays at the frequency necessary to validate the ongoing efficacy of UV-C decontamination systems, especially in healthcare settings. UV-C decontamination systems must be regularly validated because the irradiance reaching an N95 respirator can vary with UV-C light source age, environmental factors such as temperature, and setup-dependent shadowing or reflections. In particular, the material properties of nearby surfaces, such as UV-C reflectivity, have a substantial influence on the spatial pattern and magnitude of UV-C dose delivered to N95 respirators [34, 35]. Thus, even if the UV-C output or pathogen inactivation efficacy of a particular UV-C lamp or decontamination system has been rigorously characterized by the manufacturer, frequent UV-C dose measurements are a more scalable, reliable, and cost-effective method (as compared to pathogen inactivation testing) for end users to ensure the system continues to operate within specification in the particular user environment.

Despite its critical role, UV-C dose is not always calculated or reported in a standardized way [25]. Dose (energy, in J/cm^2^) is the integrated irradiance measured on a surface (W/cm^2^) over the exposure time (s). Germicidal efficacy is wavelength dependent. Thus, to compare UV-C sources with different emission spectra and to evaluate overlap between a UV-C source and the pathogen action spectrum, dose reported from polychromatic sources should weight each wavelength by its respective relative germicidal efficacy [2, 36]. Unless a detector is omnidirectional, measured UV-C dose will depend on the location and orientation of the UV-C detector with respect to the source. As a result, to ensure reproducibility, it is critical to measure and report UV-C dose along with parameters such as UV-C source, distance from and position with respect to the source, measured irradiance, and exposure time (Appendix).

Accurate dose measurements depend on the selection of an appropriate UV-C sensor. Detectors such as radiometers, dosimeters, and dose indicator strips are all used to measure and/or calculate UV-C dose. Characteristics of UV-C sensors, such as the sensor wavelength sensitivity spectrum, dynamic range, and angular response strongly affect measured values. As a result, it is important to consider the working principle of the sensor when matching a sensor to a given application. For example, radiometers can provide quantitative measurements appropriate for research or validation environments, but radiometers that do not have an ideal cosine response (e.g., those that are designed for collimated sources) will not accurately report UV-C doses from non-normal incident radiation. Additionally, angular response of UV-C sensors is often not characterized or provided. Spherical actinometric detectors relate the detector quantum yield to the dose on a surface, are widely used to calibrate physical sensors, and accurately measure dose on complex geometries. However, actinometry can be labor intensive, and the diversity of chemical transitions used in actinometry requires careful reporting for accurate measurement and reproducibility [2, 37]. Low-cost photochromic dose indicator strips can offer a straightforward colorimetric indicator of dose range, are commonly used in healthcare settings, and may facilitate implementation of UV-C decontamination across both low- and high-resource environments. However, these qualitative indicators are subject to potential pitfalls: Dose indicator strips are commonly sensitive to both UV-B and UV-C, and those designed for nonporous or low-dose applications frequently have insufficient dynamic range, saturating below the

1.0 J/cm^2^ dose required for decontamination of many N95 respirator models [14, 38]. Thus, even when the goal is simply to verify that a decontamination system is operating within specification, it is critical to understand the specificity and dynamic range of qualitative UV-C dose indicators. The ability to perform reproducible UV-C decontamination of N95 respirators, whether in the laboratory or the clinic, requires applied dose to be measured with a UV-C–specific sensor capable of measuring at least 1.0 J/cm^2^ and with maximum sensitivity aligned with the pathogen action spectrum (e.g., 260 nm). If the detector has a nonideal angular response, the beam divergence at the detector should be identical to the conditions under which the sensor was calibrated, without which measurement errors are common (Fig. 1C).

## 5 Common Measurement Pitfalls

Several common pitfalls hinder accurate measurements of UV-C wavelength and dose, some of which are listed in Table 1. One common source of error is a mismatch between the light source and detector. For example, dose measurements with a broadband sensor will collect not only germicidal UV-C but also minimally or nongermicidal wavelengths such as UV-A/B, visible, and infrared radiation, often with even greater sensitivity. Unless UV-C is specifically isolated at the sensor (e.g., with a bandpass filter), this mismatch will yield artificially high readings of UV-C dose. While different standards define different acceptable wavelength ranges of sensor sensitivity for different applications [2, 39, 40], sensors specifically used to measure UV-C should only be responsive to UV-C wavelengths between 200–300 nm and with peak response at the emission peak of the UV-C source (e.g., 254 nm for low-pressure mercury lamps) [39]. Methods used to calculate a correction factor to account for the wavelength dependence of a sensor are further described by Bolton and Linden [2]. Another common mistake is in mapping measurements of power or irradiance to dose. Since the irradiation of a UV-C source can vary over both space and time (Fig. 1B), calculations of dose determined by multiplying a single irradiance measurement by exposure time can result in overestimates or underestimates of the dose applied (as shown in Table 2). Instead, applied dose is more accurately determined by integrating irradiance measured throughout the entire exposure time, to account for fluctuations in applied irradiance.

Many of the risks associated with over- or underestimating applied dose can be managed with an understanding of the working principles of the UV-C source and detector and through adequate reporting. However, the implications of over- or underreporting UV-C dose applied to N95 respirators are wide-ranging and user-dependent, as demonstrated in Table 2. For example, if researchers studying viral inactivation overestimate the UV-C dose required to decontaminate N95 respirators (e.g., reported dose is higher than true delivered dose), then this can provide a margin of safety; however, if clinical staff overestimate the UV-C dose delivered to N95 respirators during a decontamination cycle, then this could result in incomplete decontamination and create a transmission risk. Understanding the best practices in UV-C dose measurement can help users choose the most conservative UV-C measurement approach for their application.

## 6 Best Practices for UV-C Measurements and Methods

Because UV-C dose is the key metric used to link research to implementation, understanding the best practices for characterizing and reporting UV-C dose for UV-C decontamination systems is critical for both the research and clinical communities. The measurement needs differ among communities (e.g., precise, quantitative UV-C dose readout may be valuable for researchers studying the effect of UV-C on pathogen inactivation or N95 respirator function, while clinical staff may solely need to verify that the UV-C dose applied to N95 respirators is within a specified range). However, a shared understanding of the factors impacting UV-C dose measurements is critical to allow users to accurately evaluate and implement UV-C methods for N95 respirator decontamination, in the context of current federal regulations. Here, we outline key considerations for multiple user groups when studying, evaluating, or implementing UV-C N95 respirator decontamination.

**Table 1:** Common pitfalls in UV-C dose measurement for N95 decontamination.

**Pitfall**	**Examples**
Wavelength mismatch between UV-C source and sensor	• Sensor does not detect the UV-C germicidal wavelengths because sensor is specific for UV-A/UV-B wavelengths (280-400 nm). • Sensor is broadband and measures a range of wavelengths across the UV, visible, or infrared spectrum, making it impossible to determine the UV-C-specific contribution to irradiance or dose without additional filters.
Dose indicators or sensors with insufficient dynamic range	• Photochromic dose indicator does not change color beyond 100 mJ/cm^2^. • Incident irradiance is not matched to the sensor dynamic range (e.g., irradiance is lower than the sensor noise floor or higher than the sensor saturation limit).
Dose calculated using a single measured irradiance value	• Irradiance is measured at a single time point but does not remain constant throughout the exposure period due to system-dependent variation in lamp output. • Irradiance measured at a single N95 location does not represent irradiance received across all surfaces of N95s located closer/farther from the UV-C source or closer/farther from reflective surfaces.
Sensor with limited angular response	• Incident light is only partially collected by the radiometer (e.g., due to a sensor housing or sensor field of view that is narrower than the light source output).
Dose calculated using rated UV-C lamp power	• Identical UV-C lamp bulbs with identical make, model, and power ratings may have differing output efficiencies.

**Table 2:** Importance of considering over- and under-reporting of UV-C dose.

**Problem**		**Underreporting** of UV-C dose *Measured or reported dose is lower than true delivered dose*	**Overreporting** of UV-C dose *Measured or reported dose is higher than true delivered dose*
**How?** *Example case listed; see * *Table 1* * for additional pitfalls*		Dose is calculated from a single irradiance measurement made at the start of the exposure period, but **lamp output increases throughout the exposure period as the lamp warms up.** The irradiance measurement underestimates the average irradiance over the exposure period, and thus reported (calculated) UV-C dose is lower than the true delivered dose. 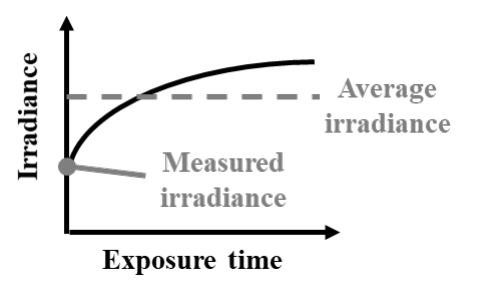	Dose is calculated from a single irradiance measurement made at the start of the exposure period, but **lamp output decreases throughout the exposure period due to changes in air temperature.** The irradiance measurement overestimates the average irradiance over the exposure period, and thus reported (calculated) UV-C dose is higher than the true delivered dose. 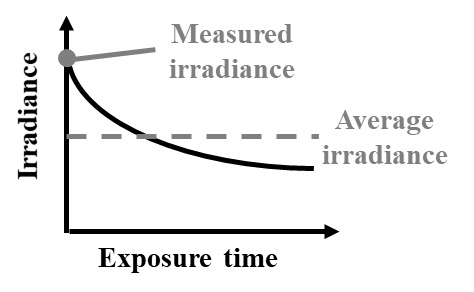
**Implications** *User is a…*	Researcher studying the impact of UV-C on N95 viral inactivation	The researcher attributes measured viral inactivation to an artificially low UV-C dose. ► Protocols based on these reported results can yield insufficient decontamination.	The researcher attributes measured viral inactivation to an artificially high UV-C dose. ► Protocols based on these reported results may recommend unnecessarily high dose and unnecessarily increase decontamination time. ► If insufficient viral inactivation was observed, report may incorrectly claim that reported dose is ineffective for N95 decontamination, potentially conflicting with other publications in which UV-C dose was measured accurately.
	Researcher studying the impact of UV-C on N95 fit & filtration	The researcher attributes measured N95 respirator damage to an artificially low UV-C dose. ► The number of decontamination cycles an N95 can withstand prior to degradation is underestimated, leading to premature disposal of scarce resources.	The researcher attributes measured N95 respirator damage to an artificially high UV-C dose. ► The UV-C dose (and number of decontamination cycles) N95s can withstand prior to degradation is overestimated, which may lead to application of damaging levels of UV-C to N95 respirators.
	Clinical staff implementing UV-C decontamination of N95s	Clinical staff underestimate the delivered dose during N95 decontamination treatments, exceeding the target dose for decontamination. ► Inaccurate decontamination protocols are perpetuated. ► N95 respirator may be damaged (if reported UV-C dose is severely underestimated).	Clinical staff overestimate the UV-C dose delivered to N95 respirators during a decontamination cycle. ► Virus may persist due to insufficient UV-C dose delivery.

*Cell color indicates the level of safety risk posed by inaccurate UV-C dose measurement in different scenarios, where yellow denotes lower risk than red.*

### 6.1 In Research

Researchers developing or studying UV-C N95 respirator decontamination systems can support safe and effective UV-C N95 respirator decontamination both in the way they perform and report UV-C measurements. In making UV-C measurements, consider the implications of over- and underestimating dose and choose the most conservative option (yellow cells in Table 2). For clinical staff to evaluate and reproducibly implement UV-C methods for N95 respirator decontamination, researchers and device manufacturers also must report in sufficient detail the way in which UV-C measurements were made [25]. Best practices, or “minimum reporting standards,” are common across scientific disciplines [41, 42]. These standards would be valuable for UV-C decontamination of N95 respirators. Standards should include physical specifications for both the UV source and the optical detector, along with necessary optical elements such as filters, diffusers, or cosine correctors. Data acquisition and analysis should also be explicitly reported, describing how dose was measured and calculated and how (or if) viral inactivation was verified. A list of suggested reporting parameters can be found in the Appendix. Thorough and standardized reporting provides a path to sidestep common pitfalls and realize the potential for UV-C to dramatically mitigate crisis-capacity conditions.

### 6.2 In Clinical Implementation

In evaluating UV-C decontamination systems: When reading and interpreting research, it is important for users to understand how UV-C dose was measured and to critically evaluate the accuracy of reported UV-C dose. To ensure N95 respirator decontamination, data should establish UV-C–induced viral inactivation on the specific N95 respirator model and in an enclosure that is comparable to that available at the workplace. To ensure that UV-C treatment does not reduce N95 respirator function, users should also assess whether preservation of respirator fit and filtration was evaluated, and they should consider how the applied UV-C dose compares to the maximum dose at which respirator integrity is expected to be maintained [11].

In implementing UV-C N95 decontamination protocols: UV-C decontamination should be used only during critical N95 shortages when in accordance with federal guidelines. UV-C dose should be regularly measured, particularly at locations receiving the highest and lowest doses, as the range of applied dose impacts decontamination efficacy and the number of times N95 respirators can be safely decontaminated prior to material degradation. The calibrated sensors used for these measurements should have narrow-band UV-C detection. Other factors that are important to consider when implementing N95 respirator decontamination and reuse:

1. High UV-C exposure, whether through a single high-dose treatment or many UV-C cycles, can degrade respirator materials and reduce filtration efficacy [11]. Due to differences in material construction, the maximum dose that an N95 respirator can withstand may be model-dependent.
2. Decontamination and multiple donning and doffing cycles can affect fit [43].
3. Shadowing and irradiation of surfaces nonperpendicular to the incident UV-C angle decrease the received dose and increase dose nonuniformity. For example, the lower viral inactivation efficacy observed on N95 facepieces with ridges has been attributed to shadowing [9]. The irradiance reaching shadowed surfaces will depend on the absorbance of the material in the optical path between the UV-C source and shadowed surface. Additionally, because irradiance depends on the angle of incident radiation [15], N95 respirator surfaces that are steeply sloped with respect to the incident light will generally receive a lower UV-C dose (Fig. 1A).
4. Soiling agents (saliva, oils) can modulate pathogen inactivation efficacy by reducing UV-C penetration into the respirator material [44, 45].
5. Viral inactivation can be N95 respirator model-dependent [9].
6. Other pathogens with lower UV-C susceptibility, especially bacterial spores, may remain active on N95 respirators even if the applied UV-C dose achieves viral inactivation [46–48].
7. Elastic straps may require a secondary decontamination method [9, 44].

Application of the appropriate UV-C wavelength and application of the appropriate dose are critical metrics for reproducible UV-C N95 respirator decontamination protocols under crisis-capacity conditions. Engaging vertically integrated teams with engineering, infection-control/sterile-processing, and clinical expertise promotes technical validation and safe processing workflows. Full consideration of the technical and practical considerations of UV-C N95 respirator decontamination is key to more safely weathering pandemic-induced crisis-capacity conditions.

**Appendix**

Recommended reporting summary for authors sharing research on UV-C decontamination of N95 respirators to support dissemination of accurate and reproducible UV-C decontamination protocols.




